# Validation and demonstration of a pericarp disc system for studying blossom-end rot of tomatoes

**DOI:** 10.1186/s13007-021-00728-3

**Published:** 2021-03-10

**Authors:** Nicholas F. Reitz, Elizabeth J. Mitcham

**Affiliations:** Department of Plant Sciences, University of California, 1106 Wickson Hall, One Shields Ave., Davis, CA 95616 USA

**Keywords:** Blossom-end rot, Calcium deficiency, Tomato, Pericarp discs, Oxidative stress

## Abstract

**Background:**

Blossom-end rot in tomatoes is often used as a model system to study fruit calcium deficiency. The study of blossom-end rot development in tomatoes has been greatly impeded by the difficulty of directly studying and applying treatments to the affected cells. This manuscript presents a novel method for studying blossom-end rot development after harvest in immature whole fruit and in pericarp discs.

**Results:**

Pericarp discs removed from the bottom pericarp of immature healthy fruit developed blossom-end rot like symptoms, corresponding to a decrease in L* value and an increase in a* value. Symptoms also developed in columella tissue, but not in stem-end pericarp tissue, similar to patterns observed during blossom-end rot development on the plant. Ascorbate oxidase and peroxidase activity, which are elevated in blossom-end rot affected fruit compared to healthy fruit, were both correlated with colorimetric measures of tissue darkening in discs. Respiration rate was higher in discs that later developed blossom-end rot symptoms, with increased respiration in asymptomatic discs on day 1 of storage being associated with symptom development on day 2. Calcium chloride and ascorbic acid treatments inhibited symptom development, demonstrating the potential of this method to provide causal evidence.

**Conclusions:**

Results indicate that symptom development in this system is consistent with blossom-end rot development with regards to location, color change, and the activity of key enzymes. This system has the potential to be used to elucidate the cause of fruit calcium deficiency and improve knowledge of the biological basis for calcium’s diverse effects on fruit.

**Supplementary Information:**

The online version contains supplementary material available at 10.1186/s13007-021-00728-3.

## Background

Blossom-end rot (BER) in tomatoes and peppers causes significant losses in the vegetable industry each year. BER is characterized by water-soaking of the tissue and cell death in the blossom-end pericarp. This is followed by blackening and sometimes drying of the affected tissue. BER occurs during fruit development prior to physiological maturity [1]. While calcium deficiency in fruit tissue and abiotic stress can increase BER, the biological mechanism of cell death remains unknown and current treatments are not fully effective.

Many previous studies on the biological drivers of BER development in tomatoes and peppers have relied on comparing measurements from BER affected fruit to healthy fruit. This approach has identified several possible causes of cell death. Comparing BER affected tomatoes grown in a low calcium hydroponic solution to healthy fruit grown with a higher calcium hydroponic solution, Mestre et al. [2] found a reduction in glutathione content, glutathione reductase activity, and catalase activity. Increased hydrogen peroxide and lipid peroxidation were also associated with BER affected fruit. Aloni et al. [3] found depleted apoplastic and symplastic ascorbic acid contents and increased apoplastic ascorbate oxidase activities in BER affected peppers compared to healthy peppers. An investigation of intracellular wash fluids by Turhan et al. [4] found increased hydrogen peroxide concentration and peroxidase activity in BER affected fruit compared to healthy fruit. While these results strongly support the trend of increased oxidative stress during blossom-end rot development, the conclusions thus far are mainly correlative in nature. Through overexpression of a vacuolar calcium importer, de Freitas et al. [5] found that increasing vacuolar calcium and decreasing apoplastic calcium increased BER development in tomatoes. These results suggest that decreased apoplastic calcium can lead to BER development, though the hypothesis that adequate apoplastic calcium incurs a protective effect has not yet been tested. Additionally, confounding factors at the whole plant, fruit, and cellular level make it difficult to determine the sequence of events leading to cell death during BER development. To eliminate these confounding factors and establish causative evidence regarding the biological cause of BER, direct manipulation of the apoplastic calcium concentration and antioxidant capacity is needed.

Pericarp discs have been used previously to study ripening in tomatoes [6, 7]. The fruit used in these experiments were at the mature green stage and the discs studied during these experiments followed ripening trends regarding physiology, visual appearance, and original tissue location. This paper describes a new method for studying BER development in immature tomatoes using the pericarp disc system.

## Results

### Symptom development in immature whole fruit after harvest

**Fig. 1:**
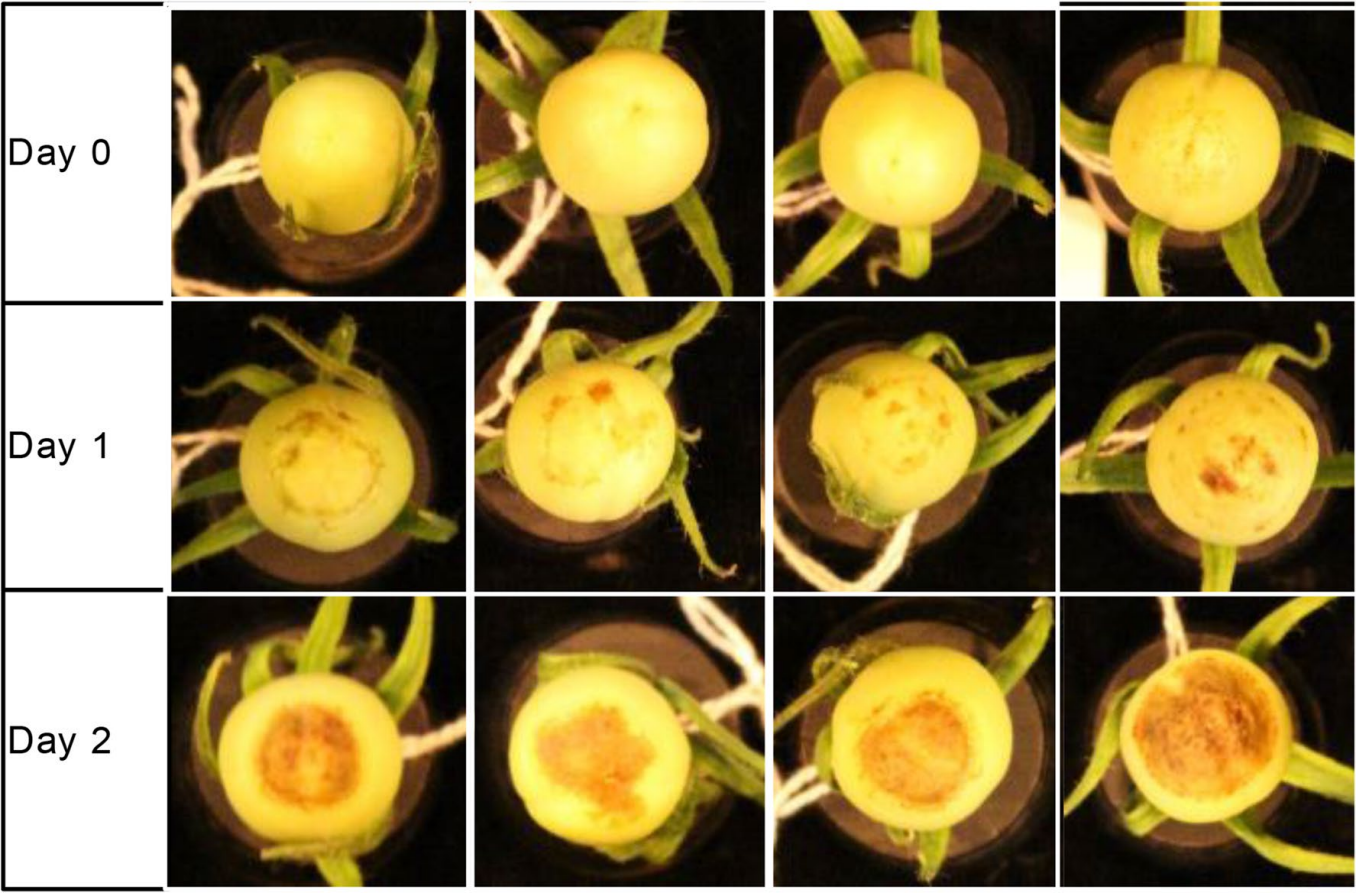
Whole HM 4885 tomatoes (n = 4 fruit) harvested immature and stored in high humidity developed blossom-end rot over 2 days of storage

Immature green tomatoes (Variety Harris Moran 4885 (HM)) showing no symptoms of BER were observed to develop BER symptoms during storage after harvest, if harvested just prior to the usual timing of BER development (approximately 8–12 days after pollination) on the plant (Fig. 1). BER development off the plant occurred within the first 3 days after harvest and the symptoms resembled BER regarding location and visual development. These results were replicated with 21-day old fruit of the HM variety and in the Rutgers variety.

### Visual development of symptoms

**Fig. 2:**
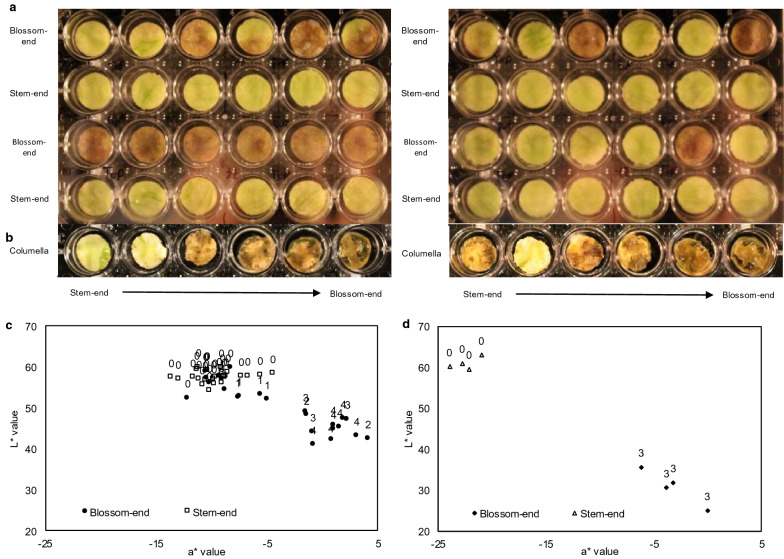
Symptom development and color analysis of pericarp and columella discs after 4 days of storage. The top images (**a**) show pericarp discs from the blossom-end (rows 1 and 3) and stem-end (rows 2 and 4) of a total of 4 fruit. Within each image, rows 1 and 2 contain discs from the same fruit, and rows 3 and 4 contain discs from the same fruit. No fruit were showing blossom-end rot symptoms prior to disc preparation. Columella discs from two fruit (**b**) were arranged with the disc taken from closest to the stem-end on the left and each subsequent disc being taken from closer to the blossom-end. Colorimetric measurements were made on the pericarp discs shown above (**a**) on day 4 of storage. Each point represents an individual disc, and data labels indicate the visual BER rating at the time of color measurement. Pericarp color of healthy stem-end tissue and BER affected blossom-end tissue was measured on 4 whole fruit that developed BER on the plant (**d**)

Pericarp discs were made from the stem-end, blossom-end, and columella tissue of immature HM tomatoes 21 days after pollination (Additional file 1: Figure S1a). During the first 12 h of storage, disc color lightened slightly and developed a “frosted” appearance as described previously [7]. In discs made from stem-end pericarp, this appearance remained the same for 4 days. In blossom-end pericarp discs, symptoms developed resembling the progression of BER development on the plant. Water soaking of the tissue was first observed on day 2 of storage, usually followed by tissue darkening. While water soaking often started on day 2, initiation of water soaking also occurred on day 3 and 4 in some blossom-end discs. Water soaking and tissue blackening was initially localized to one area of the disc, and subsequently spread to the rest of the disc, as observed in Additional file 2: Video S1. An example of these symptoms is shown in (Fig. 2a). These results were replicated in Rutgers and Ailsa Craig tomatoes (data not shown). Darkening in top discs was observed only when the discs were excessively handled for color or weight loss analysis, and was visually similar to symptom development in blossom-end discs. In columella discs, symptoms developed similarly to that of pericarp discs. Discs taken from closer to the stem-end often maintained a green-white appearance, while discs taken from near the blossom-end darkened over the 4-day storage period (Fig. 2b). In blossom-end pericarp discs made from fruit already showing BER symptoms, the blackening spread from the BER affected area to the non-affected area. HM and Ailsa Craig tomatoes produced the most consistent results (data not shown). Rutgers tomatoes were more susceptible to symptom development at sites of damage from handling in stem-end discs (data not shown).

### Colorimeter measurements

Color was measured in HM discs (Fig. 2c) and whole fruit harvested with BER symptoms (Fig. 2d). L* and a* values were used to quantify darkening and loss of green color, respectively. Visual evaluations of symptom development in discs were also completed for comparison (Additional file 1: Figure S1b). Discs exhibiting symptoms and BER tissue in whole fruit harvested with BER symptoms had lower L* values and higher a* values than stem-end discs or the stem-end of whole fruit harvested with BER symptoms. Blossom-end discs that did not develop symptoms had similar values to those of the stem-end discs.

### Weight loss in discs during storage

**Fig. 3:**
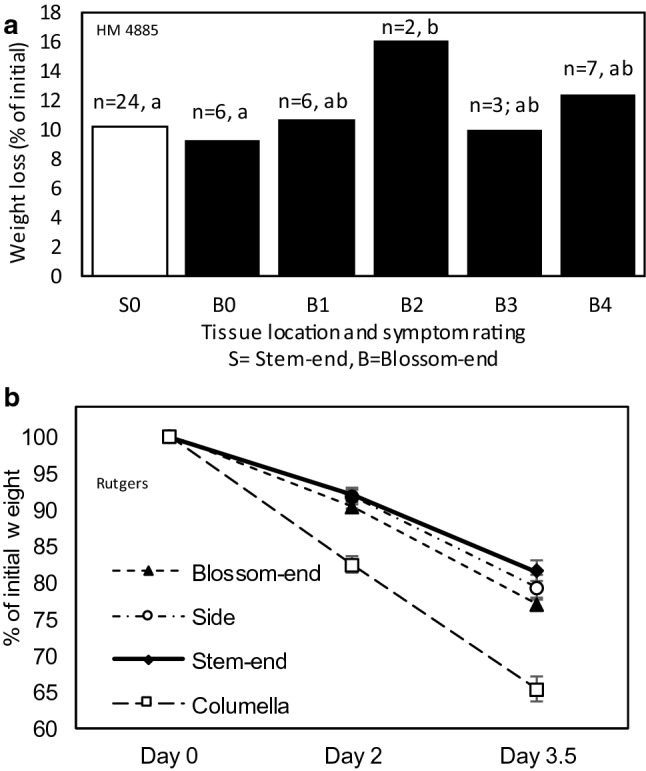
**a** Percent weight loss for stem-end discs (S0) and blossom-end discs from HM 4885 tomatoes at each visual symptom rating (B0 to B4). Data above each bar represents the sample size and means separation. Bars with the same letter were not significantly different (p > 0.05). **b**) Weight loss over 3.5 days of storage in stem-end, side, blossom-end, and columella discs (n = 12, 12, 12, and 11, respectively) from Rutgers tomatoes. Values are presented as the percent of the initial disc weight and error bars represent the standard error of the mean

HM disc weight loss over a 4-day storage period was not significantly different between stem-end discs and asymptomatic blossom-end discs (Fig. 3a). Discs with a visual symptom rating of 2 had significantly higher weight loss than asymptomatic blossom-end discs and stem-end discs. Weight loss in Rutgers discs was higher than HM discs. Weight loss for stem-end, side, and blossom-end Rutgers discs was slightly higher from day 2 to day 3.5 than day 0 to day 2 (Fig. 3b). Weight loss in columella discs was similar from day 2 to day 3.5 and day 0 to day 2.

### Enzyme analysis and color correlation

Enzyme analysis and color measurements were taken after 4 days of storage on 24 top and 24 bottom discs. A Pearson correlation analysis between enzyme activities and color measurements from HM discs showed that both pyrogallol peroxidase and ascorbate oxidase were significantly negatively correlated with L* values and positively correlated with a* values (Table 1). The mean ascorbate oxidase activity was 193.8 nmol min^−1^ g^−1^. The mean pyrogallol peroxidase activity was 11.7 μmol min^−1^ g^−1^. Both ascorbate oxidase and pyrogallol peroxidase activity means were significantly higher in blossom-end discs compared to stem-end discs.

**Table 1:** Pearson correlation analysis (top value) and p value (bottom value) between enzyme activity and color measurements from HM4885 discs (n = 48).

	L*^a^	a*^b^	Ascorbate oxidase	Pyrogallol POX
L*	1	−0.873	− 0.362	− 0.423
		< 0.0001	0.0115	0.0027
a*	− 0.873	1	0.438	0.516
	< 0.0001		0.0019	0.0002
Ascorbate oxidase	− 0.362	0.438	1	0.5075
	0.0115	0.0019		0.0002
Pyrogallol POX	− 0.423	0.516	0.508	1
	0.0027	0.0002	0.0002	

^a^L* is a measure of lightness with black at 0 and white at 100,

^b^a* is a measure of red to green coloration, with green in the negative direction and red in the positive direction

### Respiration measurements

**Fig. 4:**
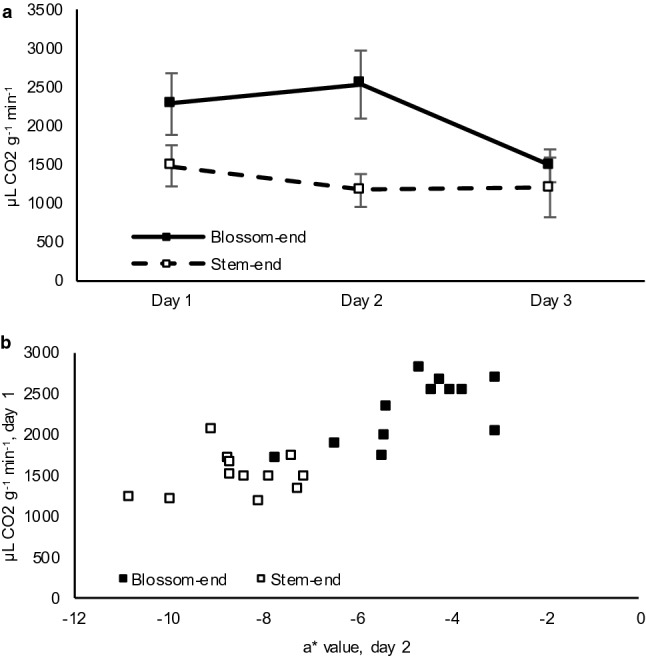
Respiration rate of HM 4885 tomato pericarp discs. Respiration rates over three days of storage (**a**) is presented as the mean of 12 discs with error bars representing the standard deviation. Respiration on day 1 compared to color analysis (a* value) on day 2 (**b**) is presented with each point representing an individual disc

Blossom-end discs had a higher mean respiration rate than stem-end discs during the first two days of storage (Fig. 4a). Respiration rates on day 1 were correlated with measurements of tissue darkening and loss of green color on day 2. The Pearson’s correlation coefficient was − 0.752 and 0.821 when comparing day 1 respiration to day 2 L* values and a* values, respectively (Fig. 4b).

### Calcium and ascorbic acid treatments

**Fig. 5:**
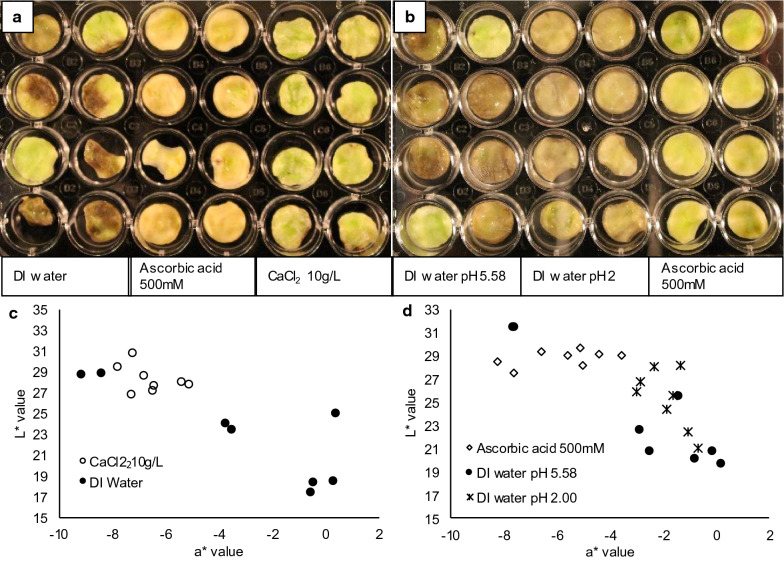
Symptom development in pericarp tissue discs 3.5 days after a 15 min soak treatment in **a** water, 500 mM ascorbic acid, or 10 g/L (90.1 mM) calcium chloride, and **b** DI water (pH 5.58), DI water (pH 2 .00), or 500 mM ascorbic acid. **a**, **b** Each show a total of 24 blossom-end discs from 4 fruit. Each treatment was applied to 2 discs from each of the 4 fruit. Colorimetric data from discs in **a** and **b** is presented in **c** and **d**, respectively

Treating discs with a 15 min soak in either 10 g/L calcium chloride or 500 mM ascorbic acid was effective in inhibiting the development of visual symptoms. Both visual evaluation (Fig. 5a and b) and color data (Fig. 5c and d) indicates that symptoms only developed in deionized (DI) water pH 5.58 and DI water pH 2.00 treated discs. Ascorbic acid treatment caused a mild loss of green color as seen in the slight increase in a* value compared to calcium treatments. Aside from this color change, ascorbic acid and calcium treated discs appeared healthy with no water soaking or darkening. Discs treated with DI water pH 2.00 showed water soaking and tissue degradation, and exhibited a gray color. Gray color development became apparent 3 days after harvest and disc formation, and became more noticeable on day 3.5, similar to the timing of tissue darkening observed in DI water-treated discs. In addition to 15 min soaking treatments, vacuum infiltration was tested as a method for inducing the uptake of treatment solutions. However, vacuum infiltration was found to induce a translucent and water-soaked appearance in all treatments, making water-soaked symptoms associated with blossom-end rot hard to identify (results not shown). The vacuum infiltration method was not pursued further.

## Discussion

### Comparison of symptom development in discs and whole fruit

Symptom development in harvested whole fruit and disc systems closely resembled symptom development in tomato fruit on the plant. Harvested whole fruit developed symptoms only at the blossom-end of the fruit. Similarly, only discs from the blossom-end of fruit developed symptoms in the pericarp disc system. Similar to trends in whole fruit [8], columella tissue taken from near the blossom-end of the fruit developed BER like symptoms while top columella tissue often did not develop symptoms.

Visual BER symptom development in whole fruit on the plant begins with water soaking of the tissue at the blossom-end, usually in one or a small number of spots. Water-soaked tissue begins to darken until it has a blackened appearance [1]. In discs, symptoms developed in a similar manner, starting with the development of a water-soaked and somewhat translucent appearance. Water-soaked areas then darkened. Drying of the affected tissue in discs was limited, likely due to the high relative humidity environment, though weight loss evidence suggests that increased water loss can occur during symptom development. Drying of the BER affected area is also common in fruit that develop BER on the plant.

### Comparison of enzyme activity

Ascorbate oxidase activity was used as a measure of the overall trend in antioxidant depletion in BER affected fruit. Maintaining ascorbic acid concentrations in fruit has been suggested as key to BER resistance and increasing ascorbic acid concentrations in vulnerable fruit tissues is a possible treatment to inhibit BER incidence [3, 4]. However, conflicting ascorbate concentrations have been reported in BER fruit compared to healthy fruit [2, 3]. Thus, direct measurement of ascorbic acid is an unreliable indicator for the state of the antioxidant system and was deemed inappropriate for the purpose of comparing BER processes in whole fruit to symptom development in discs. Alternatively, ascorbate oxidase activity has been shown to be increased in BER affected peppers compared to healthy peppers [3]. In tomato, ascorbate oxidase was found to be transcriptionally upregulated in fruit treated with the BER-inducing phytohormone gibberellin [9]. Additional file 3: Figure S2 shows ascorbate oxidase activity in healthy top, healthy bottom, and BER affected pericarp tissue from BER affected fruit, and healthy top and healthy bottom pericarp tissue from BER unaffected fruit. This data indicates that the ascorbate oxidase activity from stem-end pericarp tissue of a BER-affected fruit is similar to the stem-end and blossom-end of healthy fruit. In our pericarp disc system, ascorbate oxidase activity was significantly correlated with objective measures of tissue darkening (negatively correlated with L* values and positively correlated with a* values), suggesting that similar antioxidant depletion processes occur during symptom development in discs as in whole fruit.

Peroxidase activity was chosen as a second indicator of enzymatic similarities between symptom development in discs and BER development on the plant. Peroxidase activity measured using an aromatic electron donor was shown to be greatly increased in BER fruit compared to healthy fruit [4, 10]. This trend has been linked to increased lignification, and peroxidase trends presented by Reitz and Mitcham [10] are similar to those found for ascorbate oxidase activity in Additional file 3: Figure S2. In our disc system, peroxidase activity was significantly correlated to measures of tissue darkening (negatively correlated with L* values and positively correlated to a* values), suggesting a similar trend to that of increased peroxidase activity in BER affected whole fruit on the plant as compared to healthy fruit.

### Respiration during symptom development

Regulation of respiration is vital to plant health, with increased respiration indicating an increase in energy consumption and possibly stress. Results presented here indicate symptom development is associated with an increase in respiration rate. Furthermore, the correlation between respiration on day 1 and color measurement on day 2 indicates this increase in respiration rate occurs prior to visual symptoms. Blossom-end disc respiration decreased to near the level of the stem-end discs on day 3, possibly due to cell death or exhaustion of the available chemical energy pool. This also indicates that processes associated with blossom-end rot are likely active prior to visible symptom development.

### Calcium and ascorbic acid treatments

Calcium deficiency in soil and fruit tissue has long been associated with BER incidence [1, 11]. However, abiotic stress can also increase BER incidence, bringing into question the direct relationship between BER occurrence and calcium deficiency. We applied calcium chloride and ascorbic acid to disc tissues to test the direct effect of increased calcium and antioxidant potential on BER development. Both treatments eliminated symptom development as evaluated visually and by colorimeter.

Calcium localization within the cell is a key factor in BER incidence. De Freitas et al. [5] directly studied the effect of calcium localization at the cellular level by increasing vacuolar calcium through increased expression of the sCAX1 gene. Increased vacuolar calcium was associated with decreased apoplastic calcium and increased BER incidence. Calcium applied to the pericarp disc systems would likely diffuse into the apoplast, testing the opposite localization state compared to that of De Freitas et al. [5]. As expected, the opposite effect (inhibition of BER symptoms) was observed when calcium was applied.

Correlation of BER symptom development with increased ROS accumulation and depleted antioxidant activity has been well established in the literature. Similar to calcium localization, decreased antioxidant potential in the apoplast has been shown to be correlated with BER symptom development [2, 4, 12]. Ascorbic acid treatment of discs inhibited BER symptom development, representing the first causative evidence that increasing antioxidant potential in the apoplast incurs a protective effect against BER development.

### Potential uses, advances, and limitations

The pericarp disc system used with immature green tomatoes has multiple potential uses for future research. As demonstrated by the respiration data presented here, using this method in combination with non-destructive measurements can allow for active monitoring of the biological changes in the discs as symptoms develop. Similarly, performing destructive analyses on half of each disc while allowing the remaining half of the disc to continue the storage progression may allow for a better understanding of why some discs and tissues develop symptoms while others do not. However, care should be taken to reduce the risk of handling induced symptoms if this technique is pursued. Finally, and most beneficial, treating pericarp tissues directly can provide causative evidence for testing hypotheses that are currently supported by correlative evidence. Ascorbic acid and increased antioxidant capacity generally, have been proposed as incurring a protective effect against BER development [2–4]. Results presented here provide causative evidence to support ascorbic acid’s protective effect. Similarly, much of the evidence for calcium’s role at the tissue level has been correlative in nature. Results presented here provide the first causative evidence for calcium’s protective effect against BER development at the cellular level. This method can be used to explore the mechanisms of BER development. It would be particularly interesting to test the effect of calcium transport and receptor inhibitors. Inhibitors of enzymes involved in ROS metabolism could also be tested. Varied timing of such treatments during symptom development could also improve knowledge of the timing of events involved in BER development.

This method could also be used to screen new BER treatments before larger scale testing in the field or greenhouse. Additionally, fruit phytotoxicity of non-BER specific field treatments could also be assessed using the pericarp disc system. It is worth noting, however, that treatments with effects at the whole plant level may not have the same effects in the pericarp system. Similarly, without access to xylem or phloem nutrient sources, cell expansion in the pericarp disc system is likely halted or abnormal. Thus, any treatment that acts through regulating cellular expansion may not be effective. However, this system is very effective in assessing treatments that disrupt pathways leading to cell death during blossom-end rot development.

## Conclusions

Harvested immature whole tomato fruit and pericarp discs develop BER like symptoms during sterile, high humidity storage. Symptoms that developed in discs resembled BER symptoms in visual appearance, tissue location, and activity of key enzymes. Respiration measurements demonstrate the effectiveness of using nondestructive methods in conjunction with this pericarp disc system. Respiration measurements also indicate that cellular processes associated with symptom development are occurring prior to the emergence of visual symptoms. Additionally, results provide causative evidence of the protective effects of calcium and ascorbic acid against BER symptom development at the cellular level. This method can be used for further research into the biology and treatment of calcium deficiency disorders.

## Materials and methods

*Solanum lycopersicum* L. (var. HM 4885) seeds were obtained from Agseeds Unlimited (Woodland, CA, USA) and *Solanum lycopersicum* L. (var. Rutgers and Ailsa Craig) seeds were obtained from the Tomato Genetics Resource Center (University of California, Davis, CA, USA). This variety is recognized by the tomato industry as highly susceptible to BER. Seeds were sprouted in peat pellets using double deionized water (ddH_2_O). Approximately 2 weeks after germination, seedlings were moved to a greenhouse and transplanted into 7.6 L pots with a mixture of 1/3 peat, 1/3 sand, and 1/3 rosewood compost, augmented with 1.56 kg m^−1^ dolomite lime. Plants were irrigated daily until pots were dripping with a solution containing 150 ppm nitrogen, 50 ppm phosphorus, 200 ppm potassium, 175 ppm calcium, 55 ppm magnesium, 120 ppm sulfur, 2.5 ppm iron, 0.02 ppm copper, 0.5 ppm boron, 0.50 ppm manganese, 0.01 ppm molybdenum, 0.05 ppm zinc, and 0.02 ppm nickel. Flowers were manually pollinated and tagged. HM fruit used for whole fruit experiments were harvested 9 days after pollination. HM and Rutgers fruit used for pericarp disc experiments were harvested 21 days after pollination.

### BER development in harvested whole fruit

Whole fruit were stored blossom-end up in a closed container under a constant flow of humidified air to reduce the accumulation of ethylene and carbon dioxide. Fruit were photographed daily.

### Disc preparation

Discs were prepared from the stem-end pericarp, blossom-end pericarp, and columella tissue on the day of harvest of 21-day-old tomato fruit. Fruit were surface sterilized in approximately 150 mL of 1% sodium hypochlorite for 1 min, then thoroughly rinsed in double deionized water. A cork borer (approximately 13.5 mm) was used to excise cylindrical tissue samples. For discs made from pericarp tissue, locular and endocarp tissue was removed using a razor blade. Discs were rinsed in ddH_2_O and blotted dry on sterilized filter paper. Discs were weighed and placed skin side down in 24 well tissue culture plates (Cellstar, Greiner Bio-one, Kremsmünster, Austria), noting the original fruit and tissue location.

For discs made from columella tissue, a 13.5 mm diameter cylinder was cut from the stem-scar to the center of the blossom-end of the fruit. The blossom-end pericarp and stem-scar were removed, producing a cylinder containing columella tissue with small amounts of locular and seed tissue. This cylinder was cut into discs approximately 2 mm in height, and these were rinsed in ddH2O and blotted dry using sterile filter paper. The discs were placed in a 24 well tissue culture plate with the disc locations from the stem-end to the blossom-end preserved. Tissue culture plates were stored without lids in a closed container under a constant flow of humidified air to reduce the accumulation of ethylene and carbon dioxide.

### Symptom rating, color, and weight loss

After approximately 3.5 days, discs were scored based on their symptom development on a 0 to 5 scale, with 0 being no symptom development and 5 being complete deterioration and blackening of the tissue. Discs were turned upside down and disc color was measured by inverting a Minolta colorimeter (Konica Minolta Inc. Tokyo, Japan) and measuring the color of the pericarp through the bottom of the plate. Disc color was measured through the bottom of the plate rather than from the top of the well to decrease effects of ambient light. In respiration and treatment testing experiments, disc color was measured through the top to reduce the mechanical damage and potential contamination associated with flipping discs pericarp side down for color measurement from the bottom. Measuring from the top is recommended for future studies. Discs were then weighed, frozen in liquid nitrogen, and stored at −80 °C for subsequent enzyme analysis. To determine weight loss trends over time, 6 Rutgers discs from the stem-end pericarp, side pericarp, and blossom-end pericarp were weighed on Day 0, Day 2, and Day 3.5 in a separate experiment.

To compare color changes in discs to the color change of fruit on the plant, 4 fruit exhibiting BER symptoms were harvested. A thin layer of skin was removed from one spot within the BER affected tissue and two spots on the stem-end to expose the pericarp tissue for comparable readings with the discs. Color was measured at these spots as stated above, and the two measurements from the stem-end were averaged for each fruit to create 4 stem-end measurements and 4 blossom-end measurements.

### Enzyme extraction and activity measurement

After 4 days of storage, color was measured on 24 stem-end and 24 blossom-end discs from HM fruit. These discs were frozen and stored at − 80 °C until enzyme extraction. Discs were ground with a pestle as they thawed in a room temperature mortar with 100 mM phosphate buffer, pH 6. The resulting homogeneous mixture was then centrifuged for 20 min at 20,000*g* and 4 °C, and the supernatant was used as a crude enzyme extract. Enzyme extracts for ascorbate oxidase activity on whole fruit were prepared using the same method.

Peroxidase activity was assayed spectrophotometrically using a BioTek H1 multimode plate reader (Biotek, Winooski VT, USA). The total reaction volume of 100 μL included 70 μL of ultrapure water, 3.33 μL of 100 mM phosphate buffer, 10.66 μL of pyrogallol, 10.66 μL of enzyme extract, and 5.33 μL 0.5% H_2_O_2_. H_2_O_2_ was added last to start the reaction. The increase in absorbance at 420 nm, associated with purpurogallin formation, was monitored over 3 min. The slope of the regression line for the plot of absorbance over time was used to calculate enzyme activity on a min^−1^ g^−1^ fresh weight basis. The extinction coefficient used for the calculations was 12.0 (mg/mL)^−1^ cm^−1^.

Ascorbate oxidase was assayed using a total reaction volume of 100μL including 75.33 μL of ultrapure water, 3.33 μL of 100 mM phosphate buffer, 10.66 μL of 250 µM ascorbic acid, and 10.66 μL of enzyme extract. The change in absorbance at 290 nm over 2 min was measured, and activity on a min^−1^ g^−1^ fresh weight basis was calculated using 2.8 mM^−1^ cm^−1^ as the extinction coefficient.

### Respiration measurement

Respiration rate was measured in HM discs by sealing each of the wells in the 24 well plate with an adhesive PCR plate cover (MicroAmp, Applied Biosystems, Foster City, California, USA) and attaching an adhesive septum (Bridge Analyzers Inc. Alameda, California, USA) to the top. Samples (1 mL) were collected by inserting a syringe into the airspace above the discs. Respiration rate was measured 1, 2, and 3 days after harvest. Wells were sealed for 5–11 min. CO_2_ production (μl CO_2_ min^−1^ g^−1^ fresh weight) was measured using an infrared CO_2_ analyzer (Horiba, Irvine, CA).

### Calcium and ascorbic acid treatments

Calcium chloride and ascorbic acid treatments were applied to discs as 15 min dips. Discs were prepared from the blossom-end pericarp tissue of 4 HM fruit as stated above. After rinsing and blotting, 2 discs from each fruit (8 total) were placed in 15 mL of either 10 g/L (90.1 mM) CaCl_2_ or 500 mM ascorbic acid and shaken gently on a rotating platform for 15 min. Controls included 15 min treatments in ddH2O (pH 5.58) or ddH2O adjusted to pH 2.00 on 8 discs each from the same fruit. The discs were blotted dry on sterile filter paper and placed in a 24 well tissue culture plate. The plates were stored in a humidified container as stated above. After 3.5 days the discs were photographed and their color measured. For color measurement of the treated discs the discs were left with the pericarp tissue facing up and the colorimeter was placed directly above each well.

### Statistical analysis

Statistical analysis was carried out using SAS On Demand for Academics (SAS Institute Inc., Cary, NC, USA). Pearson’s correlation for enzyme analysis and color measurements was completed on a total of 48 discs, 24 from stem-end pericarp and 24 from blossom-end pericarp. A general linear model procedure was used to determine significant differences in weight loss and a Tukey’s honest means separation was completed. Pearson’s correlation comparing respiration on day 1 with color measurement on day 2 was completed on 24 discs, comprised of 12 stem-end discs and 12 blossom-end discs.

## Supplementary Information

**Additional file 1: Figure S1.** Sampling locations and visual symptom scale. Fruit tissues (a) used for discs included the stem-end, middle, and blossom-end pericarp, as well as columella tissue. Representative (b) discs are shown for each rating on the 0–4 visual symptom rating scale.

**Additional file 2: Video 1.** Video depicting blossom-end rot symptoms spreading during storage of pericarp discs from immature green tomatoes. The first and third rows of discs were obtained from the stem-end of fruit and the second and forth rows of discs were made from the blossom-end of fruit.

**Additional file 3: Figure S2.** Ascorbate oxidase activity in BER unaffected and BER affected whole fruit harvested 21 days after pollination. Each bar is the mean of 4 replicates, and bars with the same letter were not statistically different (p > 0.05).

## Data Availability

The datasets generated during and/or analysed during the current study are available in the Dryad database, using the following information: Reitz, Nicholas (2020), Validation and demonstration of a pericarp disc system for studying blossom-end rot of tomatoes, Dryad, Dataset, https://doi.org/10.25338/B8DP7R.
