# Supplementary material for: Validation and demonstration of a pericarp disc system for studying blossom-end rot of tomatoes
Source: Plant Methods. 2021 Mar 10;17:28. doi: 10.1186/s13007-021-00728-3 (PMC7944904; doi:10.1186/s13007-021-00728-3)
Supplement: Supplementary file 1 — Additional file 1: Figure S1. Sampling locations and visual symptom scale. Fruit tissues (a) used for discs included the stem-end, middle, and blossom-end pericarp, as well as columella tissue. Representative (b) discs are shown for each rating on the 0–4 visual symptom rating scale. [file 13007_2021_728_MOESM1_ESM.pptx]

## Slide 1
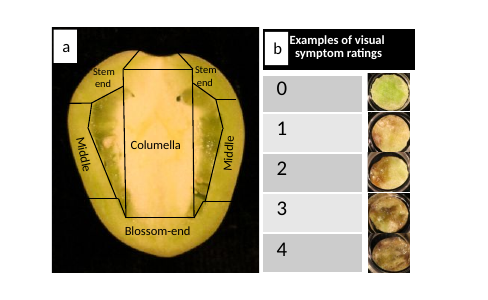

Stem
end
Stem
end
Columella
Middle
Middle
Blossom-end
a
| Examples of visual symptom ratings | Example |
| --- | --- |
| 0 | |
| 1 | |
| 2 | |
| 3 | |
| 4 | |
b
