# Supplementary material for: Validation and demonstration of a pericarp disc system for studying blossom-end rot of tomatoes
Source: Plant Methods. 2021 Mar 10;17:28. doi: 10.1186/s13007-021-00728-3 (PMC7944904; doi:10.1186/s13007-021-00728-3)
Supplement: Supplementary file 3 — Additional file 3: Figure S2. Ascorbate oxidase activity in BER unaffected and BER affected whole fruit harvested 21 days after pollination. Each bar is the mean of 4 replicates, and bars with the same letter were not statistically different (p > 0.05). [file 13007_2021_728_MOESM3_ESM.pptx]

## Slide 1
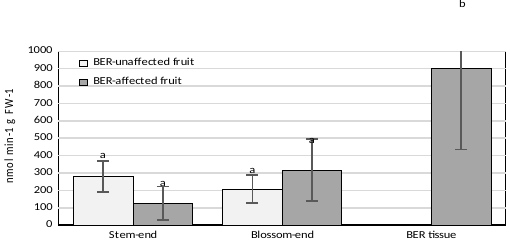

### Chart
| Category | BER-unaffected fruit | BER-affected fruit |
|---|---|---|
| Stem-end | 280.5883141248996 | 125.63655856338778 |
| Blossom-end | 207.30032162958977 | 316.18533905119244 |
| BER tissue | None | 898.3013937282233 |
